# Successful Genetic Transfection of the Colonic Protistan Parasite *Blastocystis* for Reliable Expression of Ectopic Genes

**DOI:** 10.1038/s41598-019-39094-5

**Published:** 2019-02-28

**Authors:** Feng-Jun Li, Anastasios D. Tsaousis, Tracy Purton, Vincent T. K. Chow, Cynthia Y. He, Kevin S. W. Tan

**Affiliations:** 1 Department of Microbiology and Immunology, Yong Loo Lin School of Medicine, National University of Singapore, 5 Science Drive 2, Singapore, 117545 Singapore; 2 Department of Biological Sciences, National University of Singapore, 14 Science Drive 4, Singapore, 117543 Singapore; 3 Laboratory of Molecular and Evolutionary Parasitology, RAPID group, School of Biosciences, University of Kent, Canterbury, CT2 7NJ United Kingdom

## Abstract

The microbial parasite *Blastocystis* colonizes the large intestines of numerous animal species and increasing evidence has linked *Blastocystis* infection to enteric diseases with signs and symptoms including abdominal pain, constipation, diarrhea, nausea, vomiting, and flatulence. It has also recently been reported to be an important member of the host intestinal microbiota. Despite significant advances in our understanding of *Blastocystis* cell biology and host-parasite interactions, a genetic modification tool is absent. In this study, we successfully established a robust gene delivery protocol for *Blastocystis* subtype 7 (ST7) and ectopic protein expression was further tested using a high sensitivity nano-luciferase (Nluc) reporter system, with promoter regions from several genes. Among them, a strong promoter encompassing a region upstream of the legumain 5′ UTR was identified. Using this promoter combined with the legumain 3′ UTR, which contains a conserved, precise polyadenylation signal, a robust transient transfection technique was established for the first time in *Blastocystis*. This system was validated by ectopic expression of proteins harbouring specific localization signals. The establishment of a robust, reproducible gene modification system for *Blastocystis* is a significant advance for *Blastocystis* research both *in vitro* and *in vivo*. This technique will spearhead further research to understand the parasite’s biology, its role in health and disease, along with novel ways to combat the parasite.

## Introduction

*Blastocystis* is a common enteric microbial eukaryote belonging to the Stramenopiles, infecting more than 1 billion humans along with a large variety of non-human hosts, such as farm animals, rodents, birds, reptiles and others^1,2^. Clinically, increasingly evidence has shown that its presence is associated with abdominal pain, constipation, diarrhea, nausea, vomiting, flatulence, irritable bowel syndrome-like symptoms (IBS)^3–6^, and even skin disorders^7^. Studies into the global causes of severe diarrhea, estimated to be responsible for 10.5% of overall child mortality^8^ in young children, have identified *Blastocystis* as an important pathogen, just after *Cryptosporidium* and *Giardia*^9,10^. *Blastocystis* is also an opportunistic pathogen in the context of human immunodeficiency virus (HIV)- AIDS infections^11^.

*In vitro* studies reveal that both parasite and parasite lysates exert damaging effects on intestinal epithelial cells, cause apoptosis and degradation of tight junction proteins (occludin and ZO1), resulting in increased intestinal permeability^12,13^. Adhesion of trophic forms to the intestinal epithelium and release of cysteine proteases appear to be the major triggers leading to pathogenesis, although axenic co-culture conditions and varying ratios of infection may not reflect the adhesion^14,15^ or pathogenesis *in vivo*^16,17^. Putative virulence factors identified include cysteine proteases legumain^18,19^ and cathepsin B^20^. *Blastocystis* spp. also possess immuno-modulatory effects including degradation of IgA^21^, inhibition of iNOS^22^, and upregulation of proinflammatory cytokines IL8 and GM-CSF in intestinal epithelial cells^23^, and IL1β, IL6 and TNFα in murine macrophages^24,25^. *Blastocystis* spp. may also dampen response to LPS in intestinal epithelial cells and monocytes^26^. Studies in rodent models and naturally infected pigs indicate that the parasite causes mucosal sloughing, increases goblet cell mucin, enhances intestinal permeability^17,27,28^, and induces a pro-inflammatory cytokine response with upregulation of TNFα, IFNγ and IL12^17^.

The development of suitable genetic tools including a transfection system for *Blastocystis* spp. to facilitate molecular studies will be an invaluable contribution in delineating the roles of virulence factors. Biologically, *Blastocystis* is a strict anaerobe. Although numerous intracellular organelles resembling mitochondria (mitochondrial-related organelles; MROs) exist, they are completely devoid of cytochrome enzymes^29^. These MROs have the property of both mitochondria of aerobes and hydrogenosomes of anaerobes, and are involved in various metabolic pathways including amino acid metabolism, iron-sulfur cluster biogenesis, oxygen stress response and a partial tricarboxylic acid cycle^30,31^. The organism is also capable of synthesizing various essential cellular phospholipids, and accumulates these within storage vacuoles^32,33^. Genetic modifications in which specific parasite molecules or organelles are rendered visible via reporter systems or epitope tags may lead to a better understanding of the cell biology of this poorly understood but widespread parasite.

## Results and Discussion

### Optimization of DNA delivery and transfection method

A genetic tool for manipulating gene expression in *Blastocystis* is hitherto lacking, although transient or stable transfection approaches have been successfully achieved in several parasitic protists, including trypanosomatid parasites *Trypanosoma brucei*^34,35^, *T. cruzi*^36^ and *Leishmania* spp.^36^; apicomplexan parasites *Plasmodium* spp.^37^, *Cryptosporidium* spp^38^. and *Toxoplasma gondii*^39^; parasitic excavates such as *Trichomonas vaginalis*^40^ and *Giardia intestinalis*^41,42^ and parasitic amoeba *Entamoeba histolytica*^43^. Nucleic acids were introduced by using Bio-Rad Gene Pulser Xcell or BTX ECM 630 Electroporation systems with commonly used cytomix buffer^44^ (occasionally supplemented with 2 mM ATP and 5 mM glutathione), or by using Amaxa Nucleofector device and human T-cell transfection reagent. Although the latter approach offers higher transfection efficiency in many kinds of cells, the high cost and “blackboxing” transfection programs and reagents restricted its usage in many labs.

**Figure 1:**
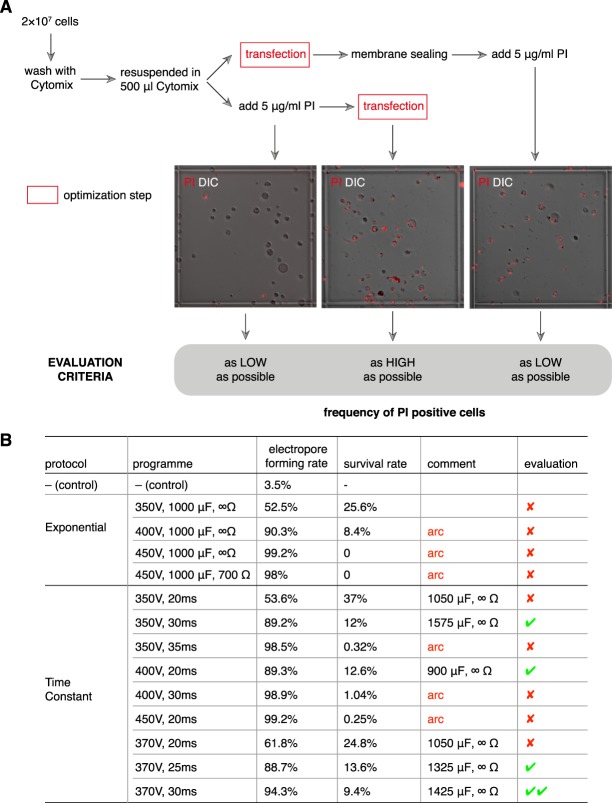
Optimization of transfection method. (**A**) Work flow and evaluation criteria for transfection optimization. (**B**) Outcomes of transfections with various programs under two different protocols (“Exponential” or “Time Constant”). “Arc” denotes arcing caused when using that particular program and protocol. The suitable programs under each protocol are indicated by green checkmarks.

A reliable gene delivery approach should introduce electropores in as many cells as possible and the pores should be sealed in sufficient number of cells to maintain the viability of cultures after transfection. Based on these criteria, we evaluated different transfection programs using the Bio-Rad Gene Pulser and cytomix supplemented with 2 mM ATP and 5 mM glutathione via an approach shown in Fig. 1A, in *Blastocystis*. To determine the electropore-forming rate, cells were washed with and resuspended in cytomix, then stained with 5 μg/ml of the cell-impermeable dye propidium iodide (PI) before transfection. The PI-positive cells were enumerated using a hemocytometer under fluorescence microscopy, and the percentage of PI-positive cells post-transfection was calculated (Fig. 1A,B). To determine the survival rate, similar batches of cells without PI staining were also subjected to electroporation and stained with PI after 12 hours’ incubation to allow membrane sealing. The PI-negative cells were counted and the survival rates were calculated (Fig. 1A,B). Two protocols (“Exponential” and “Time Constant”) and several programs with different voltage (“Exponential protocol”) or different voltage and electroporation time (“Time Constant” protocol) were evaluated. A low-voltage program was selected due to the large size of *Blastocystis* cells, which resulted in arcs when the voltage was set too high or when the electroporation time was too long (Fig. 1B). Amongst all the tested programs, one pulse with 370 V, 30 ms under the “Time Constant” protocol achieved the best result, with a 94.3% electropore-forming rate and 9.4% survival rate (Fig. 1B).

It was noteworthy that during these evaluations, only 2 × 10^7^ cells were used for each transfection. When more cells were used, the probability of arcing was higher, and shorter electroporation times should therefore be employed. In the case of 10^8^ cells per transfection, pulsing with 370 V and 20 ms resulted in excellent transfection efficiency (Ref. the followed section).

Oscillating electroporation instead of standard exponential decay electroporation was used for the slime mold *D. discoidum*^45^ and mammalian NIH 3T3 cells^46^ to increase the transformation efficiency. In this case, the Bio-Rad Gene Pulser II system is equipped with a radiofrequency module that can convert a direct electric field into an oscillatory field. This produces several oscillating pulses rather than a single decaying pulse and improves transformation efficiency by 20-fold in *D. discoidum*^45^. Since the Gene Pulser in our laboratory lacks this radiofrequency module, this approach could not be tested.

### Evaluation of NanoLuc luciferase (NLuc) expression in transfected *Blastocystis*

To drive efficient ectopic gene expression, a strong promoter and a polyadenylation signal in the 3′ UTR region are required. A crucial element of successful genetic manipulation in various protistan parasites involves the usage of species-specific, endogenous promoters that regulated a variety of housekeeping genes including actin, tubulin and GAPDH, or conserved essential genes such as histone, myosin, and enolase^38,47–50^. To test promoter activity, *Photinus pyralis* (firefly) luciferase assays are commonly used due to the extremely rapid reaction, low cost, high sensitivity and the utilization of commercially available non-radioactive substrates^51^. A new smaller and ATP-independent luciferase, deep-sea shrimp *Oplophorus gracilirostris* NanoLuc luciferase (Nluc) was developed recently by Promega. Together with its synthetic imidazopyrazinone substrate furimazine, it produces signals averaging over 150 times brighter than firefly luciferase^52^. The high stability and sensitivity of this enzyme render it an ideal tool for testing promoter activity in *Blastocystis* cells.

**Figure 2:**
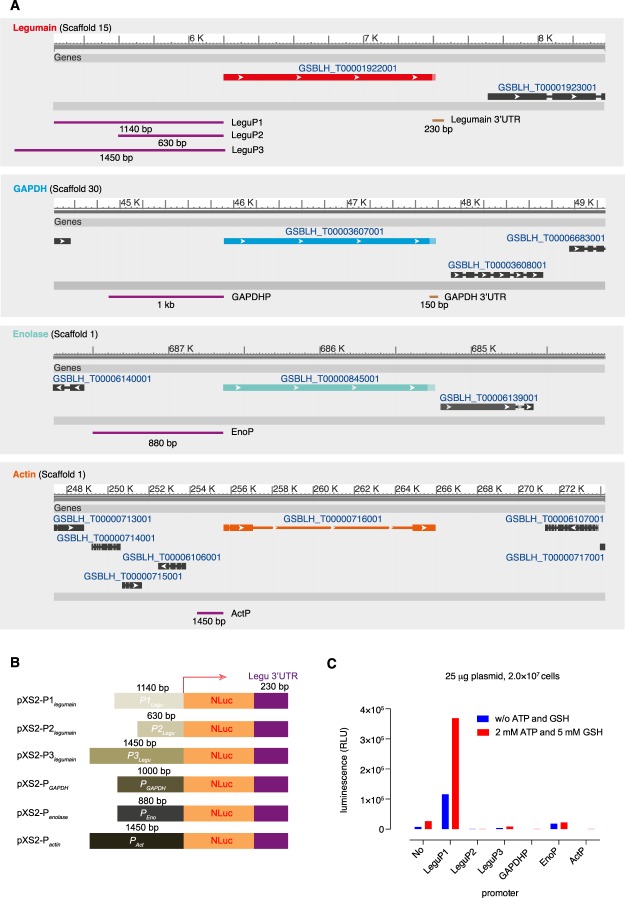
The Nluc expression controlled by different promoters. (**A**) The loci of legumain, GAPDH, enolase and actin in the corresponding chromosome scaffold. The position and size of the potential promoter and 3′ UTR regions are indicated. (**B**) The vectors used for expressing Nluc luciferase driven by different promoters and regulated by legumain 3′ UTR. (**C**) The Nluc luciferase assays for cells transfected by vectors indicated in panel B. A total of 2 × 10^7^ *Blastocystis* ST7 B cells were transfected with 25 μg of plasmid DNA using incomplete or complete cytomix buffer. One pulse with 370 V, 30 ms under “Time Constant” protocol was applied for each transfection. A transfection without plasmid DNA served as a negative control.

We replaced the YFP-tag with Nluc in the pXS2 vector that was used for ectopic expression of target genes in *T. brucei*^53^. The Nluc cassette was flanked by putative promoter sequences from the 5′ UTR regions of four different *Blastocystis* genes: two house-keeping genes GAPDH (GAPDHP) and actin (ActP), enolase (EnoP) involved in energy metabolism, and the membrane enzyme legumain that is highly expressed in *Blastocystis*^19^, together with a 230-bp potential polyadenylation signal-containing 3′ UTR downstream of the legumain coding region (Fig. 2A,B). For the potential legumain promoter, three fragments of 630, 1140 and 1450 bp upstream of the legumain (without intron) open reading frame (ORF) (designated LeguP2, P1 and P3, respectively) were tested (Fig. 2A,B). All the constructs (25 μg per transfection) were transfected into *Blastocystis* ST7 B cells (2 × 10^7^ each) using the program indicated above, and the relative Nluc luminescence was assayed using a HiDex multimodel microplate reader. Among the promoters tested, only the Nluc driven by LeguP1 produced strong luninescence, but not those by LeguP2, LeguP3, GAPDHP, EnoP and ActP (Fig. 2C). The supplementation of 2 mM ATP and 5 mM glutathione augmented the transfection efficiency about 3-fold (Fig. 2C).

**Figure 3:**
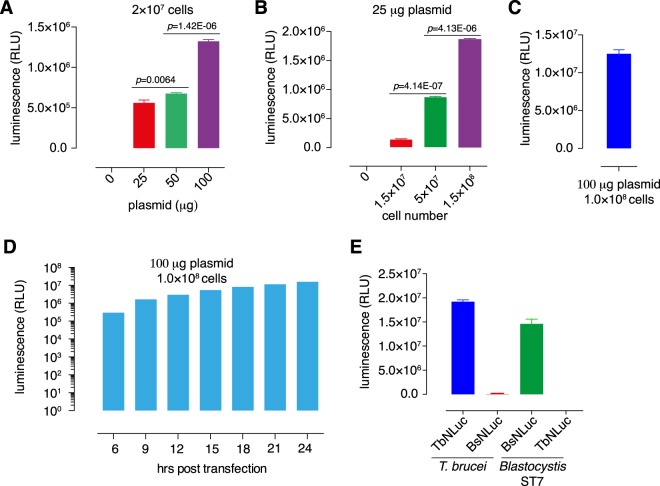
The transfection efficiency is dependent upon DNA amount and cell number. (**A**,**B**) The transfection efficiency dependent on the DNA amount (**A**) and cell number (**B**). (**C**) High transfection efficiency was achieved using 100 μg of plasmid DNA and 10^8^ cells. This condition was used in all further transfections. (**D**) The NLuc expression at different time points within 24 hrs post-transfection. (**E**) Species-specific promoter activity: PARP promoter only drives Nluc expression in *T. brucei*, while the legumain promoter drives expression in *Blastocystis* only. All of the RLU readings were shown as mean ± SD of three independent experiment and the *p* values were calculated using the 2-tailed, equal variance Student *t* test.

The Nluc luminescence correlated with the amount of DNA (Fig. 3A) and the number of parasites (Fig. 3B) used for transfection. High transfection efficiency was achieved by using 100 μg of plasmid DNA and 10^8^ cells (Fig. 3C), and this condition (370 V, 20 ms under Time Constant protocol) was used for further transfections. To test the best detection time point, the Nluc luminescence assays were conducted in transfectants with 6, 9, 12, 15, 18, 21 or 24 hours incubation post transfection. After 6 hours incubation, the expression was detectable (Fig. 3D). A relative higher expression was achieved after 15 hours’ incubation and slightly increasing at 24 hours (Fig. 3D). Nluc luminescence was also shown to be dependent upon the presence of parasite-specific promoters: the *T. brucei* PARP promoter only drives Nluc expression in trypanosomes, while the LeguP1 only drives the expression in *Blastocystis* (Fig. 3E).

**Figure 4:**
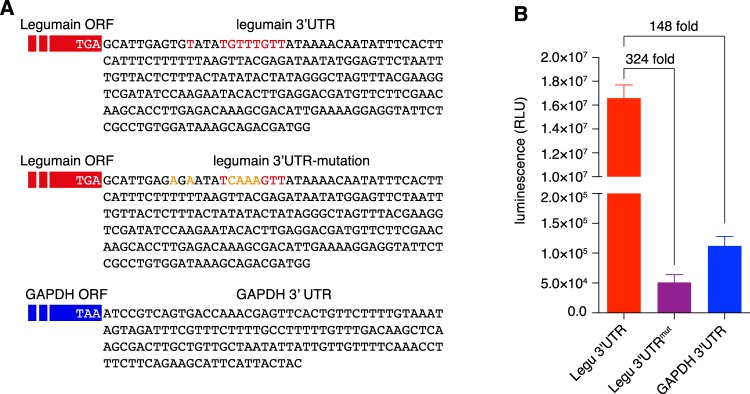
The conserved polyadenylation signal in legumain 3′ UTR contributes to the Nluc expression in two scales. (**A**) The sequences of 256-bp wild type and mutated legumain 3′ UTR and 150-bp GAPDH 3′ UTR were investigated. The conserved polyadenylation signal and potential polyadenylation sites are highlighted in red. The mutated nucleotides are highlighted in orange. (**B**) Nluc expression level was regulated by 3′ UTRs of legumain (wild type or mutated) or GAPDH. The RLU readings were shown as mean ± SD of three independent experiments.

In *Blastocystis*, an intriguing phenomenon was documented by Klimes *et al*.^54^; i.e. polyadenylation-mediated creation of termination codons occurs in about 15% of all nuclear-coding genes. In the 3′ UTR of these genes, a conserved motif TGTTTGTT is located 4 nucleotides downstream of the polyadenylation site^54^. This motif was found in legumain 3′ UTR, and a potential polyadenylation site (nucleotide T) exists 3 or 5 nucleotides upstream of the motif (Fig. 4A). To test whether this motif at the 3′ UTR region contributes to Nluc expression, two approaches were adopted. Firstly, we mutated the conserved motif to TCAAAGTT and the upstream two thymines (+3 and +5) to adenines (Fig. 4A). Secondly, we replaced the legumain 3′ UTR in the LeguP1 vector with a 150-bp GAPDH 3′ UTR fragment that lacks the conserved motif (Figs 2A and 4A). The Nluc expression level diminished 324 and 148-fold in cells transfected with vectors containing mutated legumain 3′ UTR or GAPDH 3′ UTR, respectively (Fig. 4B), indicating that the existence of a precise polyadenylation signal^54^ contributes significantly to efficient gene expression, although it is yet unknown how the expression was regulated in genes without the conserved motif in their 3′ UTRs.

There is a scarcity of information on how gene expression is controlled and regulated in *Blastocystis*. This limited our ability to select suitable promoters for ectopic gene expression. Thus far, only one promoter (LeguP1) worked well in our experience. A larger number of strong promoters need to be identified in order to expand the functionality of the existing protocol into possibilities of stable or regulatable transfection.

### Determination of the efficiency of transient transfection

**Figure 5:**
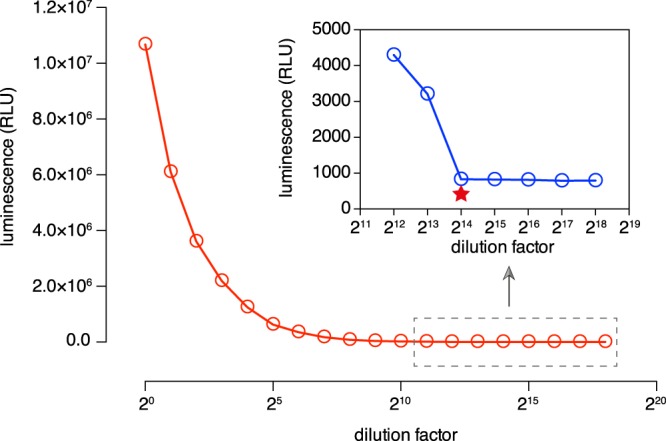
Determining the efficiency of transient transfection in *Blastocystis*. A total of 10^8^ cells were transfected with 100 μg of plasmid DNA, using “Time Constant” protocol (1 pulse of 370 V, 20 ms). The transfectants were diluted serially (2-fold dilutions) and the luminescence was monitored. After 14-steps of dilution, the RLU reading reached background level. Based on this finding, a transfection efficiency of approximately 10^−4^ was derived.

To evaluate the efficiency of transient transfection in *Blastocystis*, 10^8^ cells were transfected with 100 μg of pXS2-P_Legumain_ vector. After transfection, cells were aliquoted at 2-fold series dilution, and the luminescence was determined for each aliquot. After 14 dilution steps, the reading of Nluc luminescence decreased to background level (Fig. 5). Thus, the transfection efficacy is approximately 10^−4^ (2^14^ ÷ 10^8^).

The expression level in individual cells (reflecting the promoter strength) was not determined. However, since it was comparable with *T. brucei*, which possesses high ectopic expression level (Fig. S1), this suggested that the legumain promoter is sufficiently strong for further optimization towards stable transfection.

### Evaluation the expression of the eGFP and small epitope Ty-tagged proteins

**Figure 6:**
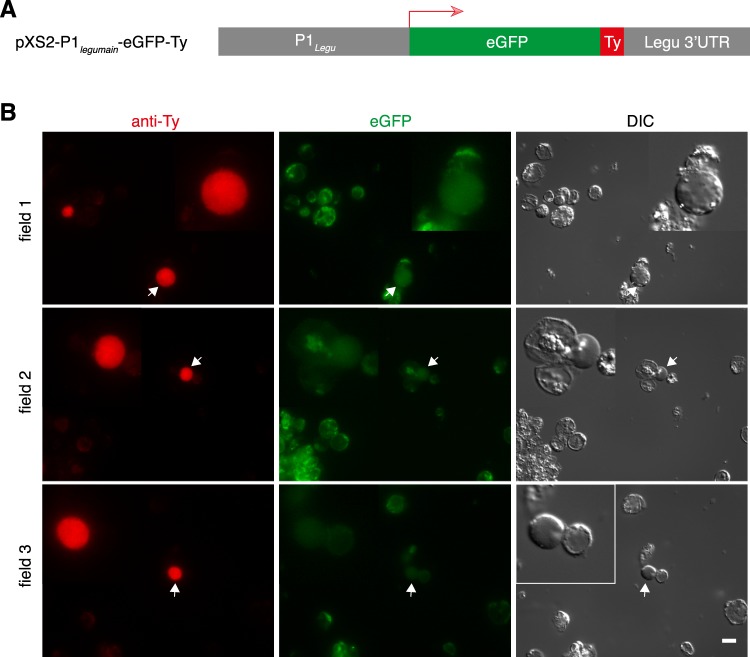
Transient expression of Ty-tagged fluorescent eGFP protein. (**A**) The plasmid used for ectopic, transient expression of eGFP in *Blastocystis* was driven by the legumain promoter and 3′ UTR. (**B**) A total of 10^8^ cells were transfected with 100 μg of plasmid DNA, using “Time Constant” protocol (1 pulse of 370 V, 20 ms). The transfectants were washed with PBS and fixed with 4% PFA. After permeabilization with 1% NP-40 in PBS, the cells were blocked with 3% BSA, and stained with mouse-anti-Ty monoclonal primary antibody followed by goat-anti-mouse IgG (Alexa Fluo 594) secondary antibody. The eGFP and Ty signals were monitored using fluorescent microscopy. Scale bar, 10 μm.

**Figure 7:**
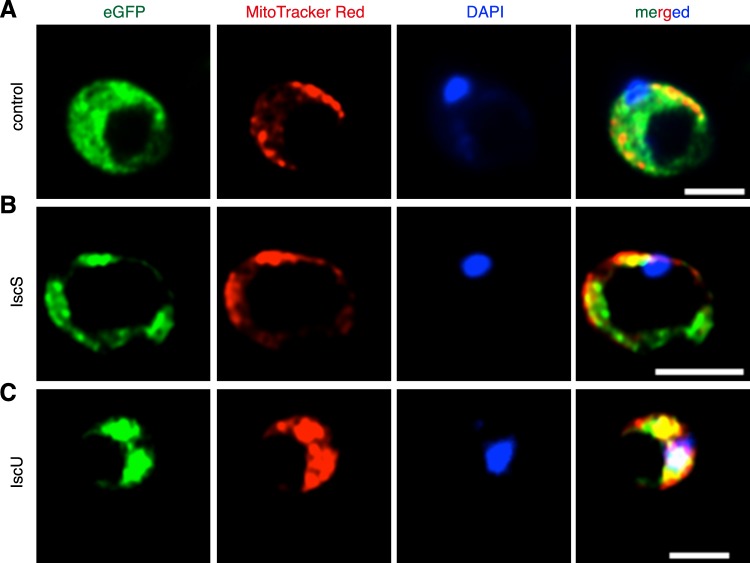
Transient expression of eGFP-tagged mitochondrial proteins. A total of 10^8^ cells were transfected with 100 μg of plasmid DNA, using “Time Constant” protocol (1 pulse of 370 V, 20 ms). After incubation for 12–16 h, transfectants were incubated under oxygen for approximately 1 h to allow for efficient folding of GFP, followed by 30 minutes of staining with MitoTracker Red. The cells were washed with PBS and fixed with 3.7% PFA. After permeabilization with 0.1% Triton-X100 in PBS, the cells were incubated with mounting medium containing DAPI. The eGFP signals were monitored using confocal microscopy. (**A**) pXS2-LeguP-eGFP-Ty-LeguUTR transfection vector was used as control, along with MitoTracker red for mitochondrial signal and DAPI for nucleus. (**B**,**C**) Constructs pXS2-LeguP-IscS-eGFP-Ty-LeguUTR and pXS2-LeguP-IscU-eGFP-Ty-LeguUTR harboring two Iron/sulfur cluster (Isc) genes showed co-localisation with MitoTracker Red for mitochondrial signal and DAPI to stain the nucleus. Scale bars, 5 μm.

To test whether the vector can be used to track protein expression by microscopy, we replaced Nluc with eGFP together with a small Ty tag^55^ (Figs 6A and S2, the full sequence of the vector can be found in Supplement Data 1). The transfectants were stained with anti-Ty antibody or MitoTracker and detected by microscopy using the eGFP channel (eGFP tag) and dsRed channel (Ty tag). Less than 1% of cells with normal cell morphology exhibited strong Ty signal (red) distributed in the whole cell, but only background level of eGFP signal was displayed (Fig. 6B). Based on previous studies, oxygen is essential for the post-translational folding of eGFP into the fluorescent chromophore^56^, which may have hindered our detection of GFP fluorescence in the anaerobic conditions of our assays. To address this limitation, cells were transfected with proteins involved in the mitochondrial iron-sulfur cluster biosynthesis, IscU and IscS, fused to eGFP and incubated in oxygen for approximately 1 h to allow for proper GFP folding. We observed punctuated concentration of the signal in intracellular structures and co-localisation with MitoTracker, suggesting a localization in *Blastocystis* MROs (Fig. 7). Both proteins were previously shown to localize in the *Blastocystis* MROs^31^, and thus our results confirm successful tagging of these proteins and pathway in the organelles. In future studies, we will employ the anaerobic cyan-green fluorescent protein evoglow Pp1 (GFPana or aFP)^57,58^ instead of eGFP.

### Towards stable transfection and potential applications

To achieve a constant expression of a target protein, several issues need to be addressed: two strong promoters to drive target and drug resistance genes; sensitive drugs for transfectant selection and incorporation of the expression cassette into the host chromosome. Till now, only one strong promoter was identified and a more comprehensive study needs to be performed to identify more robust promoters. *Blastocystis* genomes apparently do not encode components of the nonhomologous end-joining machinery, suggesting that homologous recombination is the principal mechanism for the repair of double-stranded DNA breaks^59^. Whether the recombination rate is sufficient for stable transfection selection requires further exploration.

In the post-genomic era, the generation of ectopic expression, knockdown or knockout cells using genetic tools is encouraging and will be crucial to understand the function of *Blastocystis* genes. Here we successfully developed a transient transfection system that facilitates the study of *Blastocystis* to advance in numerous new directions. To further optimize and exploit this technique, the limited knowledge of homologous recombination in *Blastocystis* is a critical area that needs to be addressed. The optimized genetic tools will be essential in studies to understand the function and localization of novel *Blastocystis* proteins as well as the orthologs of known eukaryotic and/or prokaryotic proteins that have been identified in the *Blastocystis* genome project. Notably, the proteins involved in host-microbiota-*Blastocystis* interactions, metabolic and biochemical pathways can be further characterized as these are of interest from both biomedical and evolutionary perspectives. Furthermore, such reliable expression of homologous or heterologous proteins and antigens in *Blastocystis* may facilitate potentially important *in vivo* studies into understanding the various adaptations of the parasite^31,59^ and the modulation of relevant immune responses in the gastrointestinal tract^60^.

## Methods

### Cell strain and culture

An axenized *Blastocystis* isolate ST7 B^61^ was used in this study. Cells were cultured in pre-reduced Iscove’s modified Dulbecco’s medium (IMDM) (Thermo Scientific) supplemented with 10% horse serum (Gibco) at 37 °C^62^. The culture tubes were maintained inside 2.5-liter sealed anaerobic jars with an anaerobic gas pack (Oxoid).

Procyclic *T. brucei* YTat1.1 cells were maintained in Cunningham’s medium supplemented with 15% fetal bovine serum (FBS) at 27 °C^63^.

### Vector construction

Nluc fragment was amplified from pNL1.1[Nluc] vector (#N1001, Promega) using primers Nluc-f (5′-ctagctagcatggtcttcacactcgaagatttc-3′) and Nluc-r (5′-cgggatccttacgccagaatgcgttcg-3′) and cloned into *Nhe*I/*Bam*HI-digested pXS2 vector to generate pXS2-Nluc plasmid.

The legumain and GAPDH 3′ UTRs were amplified from *Blastocystis* genome DNA using the respective primer pairs: Legu-3utr-f (5′-cgggatccgcattgagtgtatatgtttgttataaaac-3′) and Legu-3utr-r (5′-ggaattcccatcgtctgctttatccac-3′); GAPDH-utr-f (5′-cgggatccatccgtcagtgaccaaacgagt-3′) and GAPDH-utr-r (5′-ggaattcgtagtaatgaatgcttctgaagaaaggtt-3′). The 3′ UTRs were then digested by *Bam*HI/*Eco*RI and cloned into the *Bam*HI/*Eco*RI-digested pXS2-Nluc vector, resulting in pXS2-Nluc-GAPDH3UTR and pXS2-Nluc-Legu3UTR plasmids.

The LeguP1, LeguP2 and LeguP3 promoters were amplified from *Blastocystis* ST7 B genome DNA using forward primers LeguP1-f (5′-cccaagctt cgcacgtagtcagccgtt-3′), LeguP2-f (5′-cccaagcttgatcagtcggcacgttgtg-3′) and LeguP3-f (5′-cccaagcttcttgtacggattaacccatgtaaatg-3′), paired with the same reverse primer LeguP-r (5′-ctagctagcttacaaattttttatggtattatttctact-3′). Since a *Hind*III restriction site exists in the GAPDH promoter region, the GAPDHP with a mutated *Hind*III site was amplified by overlap-extension PCR using these 4 primers: GAPDHP-f1 (5′-cccaagcttgtaggcacgctctctgaatattcc-3′), GAPDHP-r1 (5′-acagaaaactgaagcatgatagagcga aaca-3′), GAPDHP-f2 (5′-tgtttcgctctatcatgcttcagttttctgt-3′) and GAPDHP-r2 (5′-ctagct agcatggaattgatatgataaagaaatcaattc-3′). EnoP and ActP promoters were amplified using the corresponding primer pairs: EnoP-f (5′-cccaagcttctactatagcagaatgtggtactgcata-3′) and EnoP-r (5′-ctagctagctctatagaaaaattttgaggatgagg-3′); ActP-f (5′-cccaagcttgcccgaccttgacttagcc-3′) and ActP-r (5′-ctagctagcatcgatgagttctttgcgtctg-3′). The amplified promoters were digested with *Hind*III/*Nhe*I and cloned into *Hind*III/*Nhe*I digested pXS2-Nluc-Legu3UTR or pXS2-Nluc-GAPDH3UTR to obtain the final vectors used for transfection (Fig. 2B).

To express the fluorescent reporter protein, an eGFP and small isotope Ty fusion fragment was amplified from pDEX-777 vector using primers eGFP-f (5′-ctagctagcagcaagggcgaggagct-3′) and eGFP-r (5′-cgggatccttagtcaagtggatcttggttagtatgg acc-3′), and digested by *Nhe*I/*Bam*HI. The digested fragment was cloned into digested pXS2 vector to generate pXS2-eGFP-Ty plasmid.

To express IscU and IscS proteins along with eGFP tag, the IscU and IscS genes were amplified from previously published *Blastocystis* clones^31^ using primers IscU-F (5′-gctagcatgtatgcattaaccagatcg-3′), IscU-R (5′-gctagcctttgacttcttttcgctctt-3′), IscS-F (5′-gctagcatgctctcccgatttagcagt-3′) and IscS-R (5′-gctagcatgggtgctcctcttgatcgc-3′) and digested by NheI. The digested fragment was cloned into the digested pXS2-LeguP-eGFP-Ty-LeguUTR plasmid.

### RNA extraction, first-strand cDNA synthesis and reverse-transcription (RT)-PCR

Total RNA was extracted from 5 × 10^7^ *Blastocystis* ST7 B cells with TRIzol Reagent (Life Technologies, 15596-026) following the manufacturer’s protocol. After treaent with RNase-free DNase I (Roche, 04 716 728 001), the first-strand cDNA was synthesized with M-MLV Reverse Transcriptase (Invitrogen, 28025-013) using the standard protocol and PCR was performed with the primers indicated above.

### Cell transfection

For transfection of *Blastocystis*, cells were cultured to log-phase and harvested by centrifugation at 1,000 *g* for 10 min at room temperature. Cells were then washed once with pre-reduced incomplete cytomix buffer (10 mM K_2_HPO_4_/KH_2_PO_4_, pH 7.6, 120 mM KCl, 0.15 mM CaCl_2_, 25 mM HEPES, 2 mM EGTA, and 5 mM MgCl_2_), and then resuspended in pre-reduced complete cytomix (incomplete cytomix supplemented with 2 mM ATP and 5 mM glutathione). These cells were ready for transfection.

*Blastocystis* cells were mixed with an appropriate amount of plasmid DNA in a 0.4-cm transfection cuvette (Bio-Rad) and subjected to 1 pulse using the Bio-Rad Gene Pulser electroporation system under the protocol and programs shown in Fig. 1B. After electroporation, cells were transferred into fresh pre-reduced culture medium and maintained at 37 °C anaerobically for 12–16 hrs.

For stable transfection in *Trypanosoma brucei*, the constructs were linearized with *Mlu*I and transfected into YTat1.1 cells using Bio-Rad Gene Pulser. The stable transformants were selected with 10 μg/ml blasticidin (Invitrogen, R210-01).

### Nluc luciferase assay

The Nluc luminescence was monitored using Nano-Glo Luciferase Assay System (Promega) following the protocol provided. Briefly, the cells were collected and lysed using 1 × Luciferase Cell Culture Lysis reagent (25 mM Tris-phosphate (pH 7.8), 2 mM DTT, 2 mM 1,2-diaminocyclohexane-N,N,N′,N′-tetraacetic acid, 10% glycerol, 1% Triton X-100). Cell lysate was mixed with equal volume of Nano-Glo Luciferase Assay reagents and the relative luminescence units (RLU) was measured using Hidex Sense multimode microplate reader (Hidex).

### Immunofluorescent microscopy

*Blastocystis* ST7 B cells were washed with and resuspended in PBS. Cells were fixed with 4% paraformaldehyde (PFA, Sigma, P6148) in PBS at room temperature for 30 min, permeabilized with 1% NP-40 (Igepal CA-630, Sigma, I8896) and blocked with 3% BSA (Sigma, A7906) before antibody staining. Monoclonal anti-Ty^55^ (a gift from Prof. Keith Gull, University of Oxford) was used at 1:100 to label eGFP-Ty, followed by staining with goat-anti-mouse IgG H&L (Alexa Fluor 594) secondary antibody at 1:2000. Cells were then mounted onto slides and observed using an Axioplan2 inverted microscope (Carl Zeiss MicroImaging, Germany) equipped with a CoolSNAP HQ^2^ camera (Photometrics, USA) and a Plan-Apochromat 63×/1.40 oil DIC objective.

### Statistical analysis

For nanoluciferase (NLuc) assay, three independent transfections were performed and relative luminescence unit (RLU) readings were shown as mean ± SD. Statistical analyses were performed using the 2-tailed, equal variance Student *t* test.

## Supplementary information

Supplementary Information
