# Supplementary material for: Successful Genetic Transfection of the Colonic Protistan Parasite Blastocystis for Reliable Expression of Ectopic Genes
Source: Sci Rep. 2019 Feb 28;9:3159. doi: 10.1038/s41598-019-39094-5 (PMC6395660; doi:10.1038/s41598-019-39094-5)
Supplement: Supplementary file 1 — Supplementary Information [file 41598_2019_39094_MOESM1_ESM.pdf]

Supplementary Information

Successful Genetic Transfection of the Colonic Protistan Parasite *Blastocystis* for Reliable Expression of Ectopic Genes

Feng-Jun Li[a,b], Anastasios D. Tsaousis[c], Tracy Purton[c], Vincent T.K. Chow[a], Cynthia Y. He[b#], Kevin S.W. Tan[a#]

[a]Department of Microbiology and Immunology, Yong Loo Lin School of Medicine, National University of Singapore, 5 Science Drive 2, Singapore 117545

[b]Department of Biological Sciences, National University of Singapore, 15 Science Drive 4, Singapore 117543

[c]Laboratory of Molecular and Evolutionary Parasitology, RAPID group, School of Biosciences, University of Kent, Canterbury, CT2 7NJ, United Kingdom

Running Head: Genetic Modification of *Blastocystis*

[#]Address correspondence to Cynthia Y He, dbshyc@nus.edu.sg or Kevin SW Tan, mictank@nus.edu.sg.

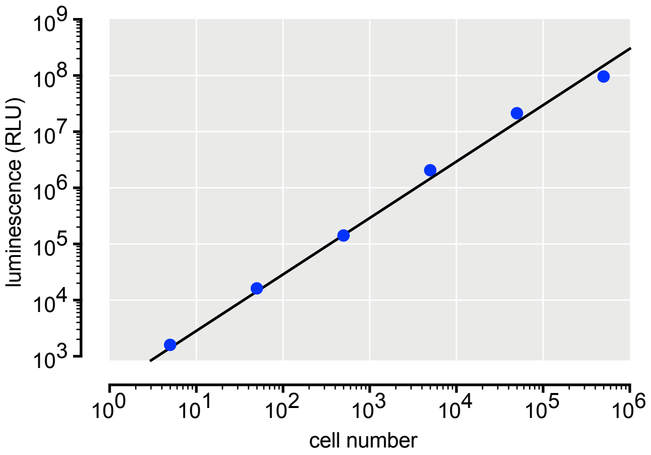

Fig. S1 Nluc luciferase expression level in *Trypanosoma brucei*. pXS2-Nluc/bla vector was transfected into *T. brucei* cells, and the stable transfectants were selected by adding 10 µg/ml blasticidin. The Nluc luminescence from 50, 500, 5,000, 5×10^4, 5×10^5 or 5×10^6 stable transformants was measured.

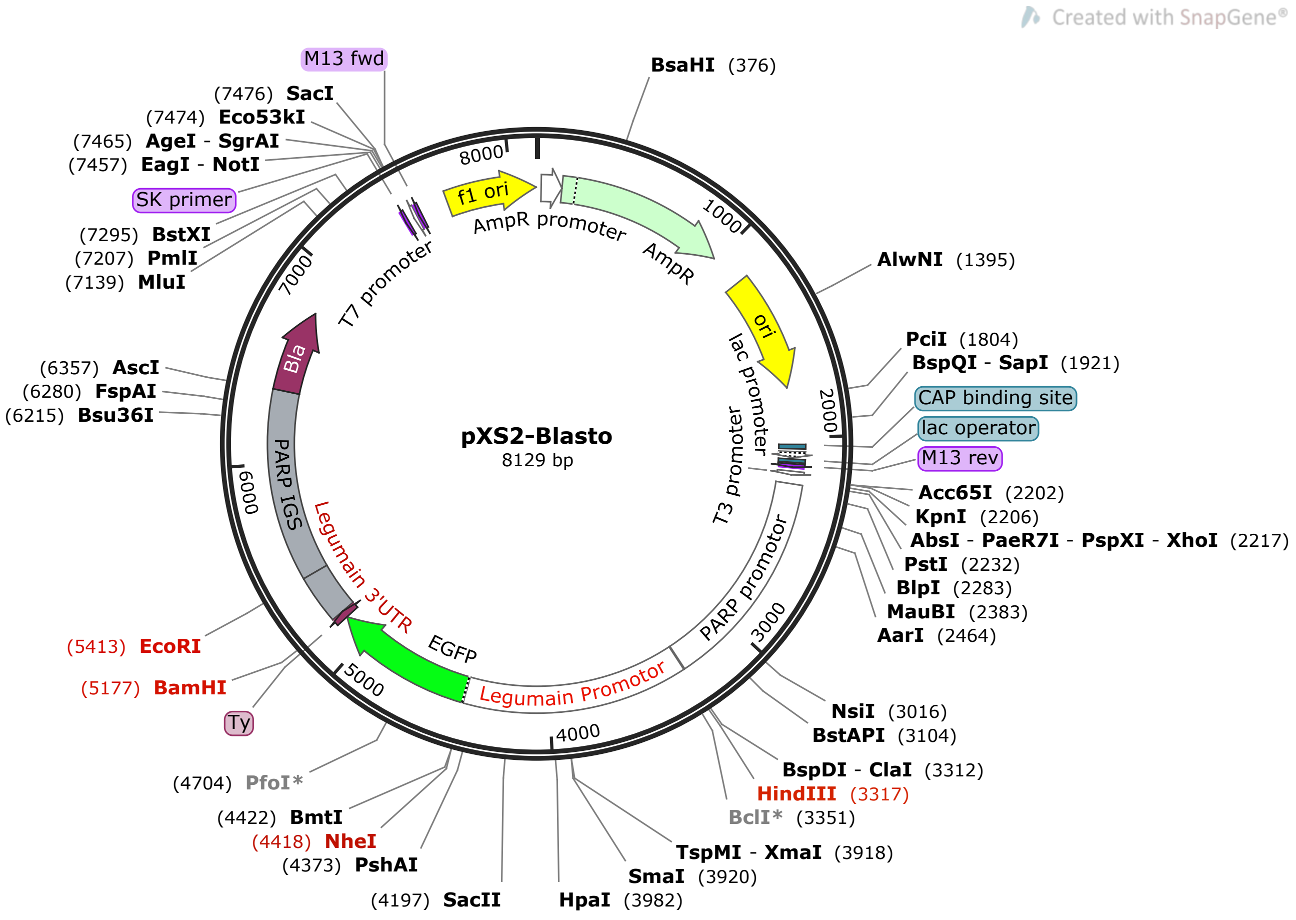

Fig. S2 The map of pXS2 vector in this manuscript for the expression of N-terminal eGFP-Ty-tagged target proteins. The map was created with SnapGene.

Supplementary Data 1: The full sequence of vector for expression of N-terminus eGFP-Ty-tagged target proteins.

```
LOCUS       Exported                8106 bp ds-DNA     circular SYN 30-APR-2018
DEFINITION  synthetic circular DNA.
ACCESSION   .
VERSION     .
KEYWORDS    pXS2-Blasto
SOURCE      synthetic DNA construct
  ORGANISM  synthetic DNA construct
REFERENCE   1  (bases 1 to 8106)
  AUTHORS   .
  TITLE     Direct Submission
  JOURNAL   Exported May 3, 2018 from SnapGene Viewer 4.1.7
            http://www.snapgene.com
FEATURES             Location/Qualifiers
     source          1..8106
                     /organism="synthetic DNA construct"
                     /mol_type="other DNA"
     promoter        24..128
                     /gene="bla"
                     /label=AmpR promoter
     CDS             129..989
                     /codon_start=1
                     /gene="bla"
                     /product="beta-lactamase"
                     /label=AmpR
                     /note="confers resistance to ampicillin, carbenicillin, and
                     related antibiotics"
                     /
translation="MSIQHFRVALIPFFAAFCLPVFAHPETLVKVKDAEDQLGARVGYIELDLNSGKILESFRPEERF
PMMSTFKVLLCGAVLSRIDAGQEQLGRRIHYSQNDLVEYSPVTEKHLTDGMTVRELCSAAITMSDNTAANLLL
TTIGGPKELTAFLHNMGDHVTRLDRWEPELNEAIPNDERDTTMPVAMATTLRKLLTGELLTLASRQQLIDWME
ADKVAGPLLRSALPAGWFIADKSGAGERGSRGIIAALGPDGKPSRIVVIYTTGSQATMDERNRQIAEIGASLI
KHW"
     rep_origin      1160..1748
                     /direction=RIGHT
                     /label=ori
                     /note="high-copy-number ColE1/pMB1/pBR322/pUC origin of
                     replication"
     protein_bind    2036..2057
                     /label=CAP binding site
                     /bound_moiety="E. coli catabolite activator protein"
                     /note="CAP binding activates transcription in the presence
                     of cAMP."
     promoter        2072..2102
                     /label=lac promoter
                     /note="promoter for the E. coli lac operon"
     protein_bind    2110..2126
                     /label=lac operator
                     /bound_moiety="lac repressor encoded by lacI"
                     /note="The lac repressor binds to the lac operator to
                     inhibit transcription in E. coli. This inhibition can be
                     relieved by adding lactose or
                     isopropyl-beta-D-thiogalactopyranoside (IPTG)."
```

```
    primer_bind     2134..2150
                    /label=M13 rev
                    /note="common sequencing primer, one of multiple similar
                    variants"
    promoter        2171..2189
                    /label=T3 promoter
                    /note="promoter for bacteriophage T3 RNA polymerase"
    promoter        2234..3311
                    /label=PARP promotor
    promoter        3317..4423
                    /label=Legumain Promotor
    CDS             4424..5140
                    /codon_start=1
                    /product="enhanced GFP"
                    /label=EGFP
                    /note="mammalian codon-optimized"
                    /
translation="MVSKGEELFTGVVPILVELDGDVNGHKFSVSGEGEGDATYGKLTLKFICTTGKLPVPWPTLVTT
LTYGVQCFSRYPDHMKQHDFFKSAMPEGYVQERTIFFKDDGNYKTRAEVKFEGDTLVNRIELKGIDFKEDGNI
LGHKLEYNYNSHNVYIMADKQKNGIKVNFKIRHNIEDGSVQLADHYQQNTPIGDGPVLLPDNHYLSTQSALSK
DPNEKRDHMVLLEFVTAAGITLGMDELYK"
    CDS             5141..5176
                    /codon_start=1
                    /label=Ty
                    /translation="MEVHTNQDPLD"
    3'UTR           5177..5418
                    /label=Legumain 3'UTR
    3'UTR           5419..6363
                    /label=PARP IGS
    CDS             6364..6785
                    /codon_start=1
                    /label=Bla
                    /
translation="MAKPLSQEESTLIERATATINSIPISEDYSVASAALSSDGRIFTGVNVYHFTGGPCAELVVLGT
AAAAAAGNLTCIVAIGNENRGILSPCGRCRQVLLDLHPGIKAIVKDSDGQPTAVGIRELLPSGYVWEG*LINP
SN"
    primer_bind     complement(7439..7455)
                    /label=SK primer
                    /note="common sequencing primer, one of multiple similar
                    variants"
    promoter        complement(7486..7504)
                    /label=T7 promoter
                    /note="promoter for bacteriophage T7 RNA polymerase"
    primer_bind     complement(7514..7530)
                    /label=M13 fwd
                    /note="common sequencing primer, one of multiple similar
                    variants"
    rep_origin      7672..8106
                    /direction=RIGHT
                    /label=f1 ori
                    /note="f1 bacteriophage origin of replication; arrow
                    indicates direction of (+) strand synthesis"
ORIGIN
        1 gtggcacttt tcggggaaat gtgcgcggaa cccctatttg tttatttttc taaatacatt
       61 caaatatgta tccgctcatg agacaataac cctgataaat gcttcaataa tattgaaaaa
      121 ggaagagtat gagtattcaa catttccgtg tcgcccttat tccctttttt gcggcatttt
      181 gccttcctgt ttttgctcac ccagaaacgc tggtgaaagt aaaagatgct gaagatcagt
      241 tgggtgcacg agtgggttac atcgaactgg atctcaacag cggtaagatc cttgagagtt
      301 ttcgccccga agaacgtttt ccaatgatga gcacttttaa agttctgcta tgtggcgcgg
```

361 tattatcccg tattgacgcc gggcaagagc aactcggtcg ccgcatacac tattctcaga
421 atgacttggt tgagtactca ccagtcacag aaaagcatct tacggatggc atgacagtaa
481 gagaattatg cagtgctgcc ataaccatga gtgataacac tgcggccaac ttacttctga
541 caacgatcgg aggaccgaag gagctaaccg cttttttgca caacatgggg gatcatgtaa
601 ctcgccttga tcgttgggaa ccggagctga atgaagccat accaaacgac gagcgtgaca
661 ccacgatgcc tgtagcaatg gcaacaacgt tgcgcaaact attaactggc gaactactta
721 ctctagcttc ccggcaacaa ttaatagact ggatggaggc ggataaagtt gcaggaccac
781 ttctgcgctc ggcccttccg gctggctggt ttattgctga taaatctgga gccggtgagc
841 gtgggtctcg cggtatcatt gcagcactgg ggccagatgg taagccctcc cgtatcgtag
901 ttatctacac gacggggagt caggcaacta tggatgaacg aaatagacag atcgctgaga
961 taggtgcctc actgattaag cattggtaac tgtcagacca agtttactca tatatacttt
1021 agattgattt aaaacttcat ttttaattta aaaggatcta ggtgaagatc ctttttgata
1081 atctcatgac caaaatccct taacgtgagt tttcgttcca ctgagcgtca gaccccgtag
1141 aaaagatcaa aggatcttct tgagatcctt tttttctgcg cgtaatctgc tgcttgcaaa
1201 caaaaaaacc accgctacca gcggtggttt gtttgccgga tcaagagcta ccaactcttt
1261 ttccgaaggt aactggcttc agcagagcgc agataccaaa tactgtcctt ctagtgtagc
1321 cgtagttagg ccaccacttc aagaactctg tagcaccgcc tacatacctc gctctgctaa
1381 tcctgttacc agtggctgct gccagtggcg ataagtcgtg tcttaccggg ttggactcaa
1441 gacgatagtt accggataag gcgcagcggt cgggctgaac ggggggttcg tgcacacagc
1501 ccagcttgga gcgaacgacc tacaccgaac tgagatacct acagcgtgag ctatgagaaa
1561 gcgccacgct tcccgaaggg agaaaggcgg acaggtatcc ggtaagcggc agggtcggaa
1621 caggagagcg cacgagggag cttccagggg gaaacgcctg gtatctttat agtcctgtcg
1681 ggtttcgcca cctctgactt gagcgtcgat ttttgtgatg ctcgtcaggg gggcggagcc
1741 tatggaaaaa cgccagcaac gcggcctttt tacggttcct ggccttttgc tggccttttg
1801 ctcacatgtt ctttcctgcg ttatcccctg attctgtgga taaccgtatt accgcctttg
1861 agtgagctga taccgctcgc cgcagccgaa cgaccgagcg cagcgagtca gtgagcgagg
1921 aagcggaaga gcgcccaata cgcaaaccgc ctctccccgc gcgttggccg attcattaat
1981 gcagctggca cgacaggttt cccgactgga aagcgggcag tgagcgcaac gcaattaatg
2041 tgagttagct cactcattag gcaccccagg ctttacactt tatgcttccg gctcgtatgt
2101 tgtgtggaat tgtgagcgga taacaatttc acacaggaaa cagctatgac catgattacg
2161 ccaagcgcgc aattaaccct cactaaaggg aacaaaagct gggtaccggg ccccccctcg
2221 aggtgggctg cagcttggca gcccaataaa ccacgcagat gtggtcccat gttcaggctc
2281 cgctgagcgt gggtcttcgt ttccccaaag cgcgaacatc cttaaccaag cccaacgcat
2341 cctggtaggt cattgtgccg acggaatttc aaccaatcag gcgcgcgcgc cacggttccc
2401 accacggcaa aagctcattg ggagtcgcgt gcccaccgaa ggtgccccct tcatatgccg
2461 gtgtgtgtgg gggcaggtgt cggctcgttt tattggtgag tcggcccacc aacgccgccg
2521 atgtgccgag ttgcgggacc gttatccgcc gaaattggtt ttaatttctg ctagttctcg
2581 ttgagagcgc atgaaaggaa aactttacca gcgttctcca tcagccgtcg tgctgaaggg
2641 ctttcagact tcttcgatgc ccggtgtttc gtagcaaaac ataccgctgc cggcctaaat
2701 gccgctgatt caccacaaag tgcttttcag tgcccccaaa ctggattgcc aaacttctga
2761 caagtgtatt gttccgagta tggtgagcga atcccttctc tattatcaat tctcgaaaac
2821 tcttcgggag tcggggccca cggaaccgag gggcggatg aggaagaggg ggaaacaaca
2881 attccgcacc gcgaacaggc accgctcccc tggacctctc cctcagatgt gaggtgcagt
2941 cagcctttgt tgtcattggg gttaagcgga aaggtgtgtg ccagtaggtt gtgaggtgaa
3001 agcgttttca gatgcatagt gagcttaatg tccttttcac agtatatcat gtctgatagg
3061 tatctcttaa cagtatagtt gagtactagt caatagtgca ttttgtgcaa aatgtccata
3121 ttgtggaagt gatatggttg ttttgtgctg ttccgtgtct ctgggtgggc gtgcattgaa
3181 aatagggtt attaggtgag tactgagttt aagatgttct cgtgatcgct gcacgcgcct
3241 tcgagttttt tttcctttta cccatttttt tcaacttgaa gacttcaatt acaccaaaaa
3301 gtagatcggt atcgataagc ttcgcacgta gtcagccgtt ggatcgtcaa tgatcatcgt
3361 ggtgagcagg ttgttgttct ggttggcaat cttcaggtcc gatgatcgca cgatcgtcga
3421 gtagacctga atcacttggc gatttcgcgt gatatcaatc acgttcgatc cactgaacga
3481 agcgggtaga atgaccgttc gtccatcgaa gccgaggatc tcacgaatgt caggtgcatc
3541 tgtgaaatca atggtgtgtg gggactgaat ccagatacag ccgaacgacg aggcattcgt
3601 cgatatggag aacagcgtgt cagtcatggt attgagcaag gcaatgatct cactgatcgt
3661 gtaataacca gcagggattg tcacagtctc gttattgctg ttagtaatca cattcgtcgt
3721 gtccacgttg ttgaacatcg aaccattagg atgaaacaac acagtcatgg aagtgatata
3781 gggattcttg tacttgtacg gattaaccca tgtaaatgag ccgttggtag gtgagccttg
3841 aataagcatg gagtagtgca tggtctttct ctctttacct tagtcaacaa gacaatgggg
3901 aggaatcgaa gattcctccc ggggcaacgt acgttgcccc atcacaccca tggataccat

3961 ggcctacaat ggaagtcagg ttaacattgc agaaatttgc cgaaattggc agccatgggt
4021 ttccgcgcgt ttcttgttat ggacatcgag gtgagcaggt agacaatggc agaaaatggt
4081 ggaaatggca gtcatgggtt tccgcgcgtt tcttgttatg gacatcgagg tgagcaggta
4141 gacaatggca gaaaatggcc gaaaatggtc gaaaaatggt cgaaaaaagt tcaccgcggt
4201 tatttggtag aaaatggatt ttcgcgcatc tatcgccttt tccctactac taccctaaag
4261 gcagaacgca ttctacaaaa tgaatttcag ataacctttg taattacgga gatagatccg
4321 taaaaatgtg ataaccacgg cagaatataa gtctattctg cgtgctgcga cctgcgtcat
4381 caatctaagt agaaataata ccataaaaaa tttgtaagct agcatggtga gcaagggcga
4441 ggagctgttc accggggtgg tgcccatcct ggtcgagctg gacggcgacg taaacggcca
4501 caagttcagc gtgtccggcg agggcgaggg cgatgccacc tacggcaagc tgaccctgaa
4561 gttcatctgc accaccggca agctgcccgt gccctggccc accctcgtga ccaccctgac
4621 ctacggcgtg cagtgcttca gccgctaccc cgaccacatg aagcagcacg acttcttcaa
4681 gtccgccatg cccgaaggct acgtccagga gcgcaccatc ttcttcaagg acgacggcaa
4741 ctacaagacc cgcgccgagg tgaagttcga gggcgacacc ctggtgaacc gcatcgagct
4801 gaagggcatc gacttcaagg aggacggcaa catcctgggg cacaagctgg agtacaacta
4861 caacagccac aacgtctata tcatggccga caagcagaag aacggcatca aggtgaactt
4921 caagatccgc cacaacatcg aggacggcag cgtgcagctc gccgaccact accagcagaa
4981 cacccccatc ggcgacggcc ccgtgctgct gcccgacaac cactacctga gcacccagtc
5041 cgccctgagc aaagacccca acgagaagcg cgatcacatg gtcctgctgg agttcgtgac
5101 cgccgccggg atcactctcg gcatggacga gctgtacaag atggaggtcc atactaacca
5161 agatccactt gactaaggat ccgcattgag tgtatatgtt tgttataaaa caatatttca
5221 cttcatttct tttttaagtt acgagataat atggagttct aatttgttac tctttactat
5281 atactatagg gctagtttac gaaggtcgat atccaagaat acacttgagg acgatgttct
5341 tcgaacaagc accttgagac aaagcgacat tgaaaaggag gtattctcgc ctgtggataa
5401 agcagacgat gggaattcta agcggatgca agcgtgtaaa gcgcctcgga ggaacgaaac
5461 cctttgaaaa ggttcctttc atttatatcg cctccatatg gtgcatcgtg tttgtttcct
5521 gctgtttctt gtaaaacaag tgtggacatt catttaatat tttttcgtta tattttttttg
5581 gtgacatcct ttctaatgcc ttattaacca tcgcctgaga cccacagccc tgtagatttc
5641 tgtgatgttt cggttgcgta ttccataatt ttaagcgttt cacttctatt ttttttcatt
5701 cctttgaatt tggatcttaa aattattatt ggtgccttgt gttattgtgc gtgctgcgtg
5761 tgaatttggt gctctgcctt ttaatttgtt ggatgagcta tttcattaat ttttttgcct
5821 tctcttcttt tgggttcgaa taatagttcc ttctaaacct tcaggccaga aatgggaaac
5881 aagtgttaga acggccaact tgggcgaagg ggtctgcatg ttgctttatt tcattggtgg
5941 ttttatgtgc agtgtttccc tgcttcagat gggccccgca gctgaagttg tttcgctgcc
6001 ctgcccccca ctgctgcgtt tctgctggag aaacgatgtt ggaggttgac gggtgtgtgc
6061 ttttgaagac tttgtatgaa tatggaagga agctcgcagc ggttactaca ggcagttaag
6121 ggctgagtgc tgtgcctttt ctattgcctc tccccctctt gatctattct tgtcgggaga
6181 gtgatttatt tatccgagtt tcggttaatt gtcccttagg gatgaaaggc acctcagaga
6241 tgagagggca atgcaacgct ggttatccca gttgatgtgc gcattcatat ataccatttc
6301 cccatttttt tcaacttgaa gacttcaatt acaccaaaaa gtaaaattcc ccatcggcgc
6361 gccatggcca agcctttgtc tcaagaagaa tccaccctca ttgaaagagc aacggctaca
6421 atcaacagca tccccatctc tgaagactac agcgtcgcca gcgcagctct ctctagcgac
6481 ggccgcatct tcactggtgt caatgtatat cattttactg gggaccttg tgcagaactc
6541 gtggtgctgg gcactgctgc tgctgcggca gctggcaacc tgacttgtat cgtcgcgatc
6601 ggaaatgaga acaggggcat cttgagcccc tgcggacggt gccgacaggt gcttctcgat
6661 ctgcatcctg ggatcaaagc catagtgaag gacagtgatg gacagccgac ggcagttggg
6721 attcgtgaat tgctgccctc tggttatgtg tgggagggct aattaattaa cccatcgaat
6781 taattcacgg aggcagagtc caacatgaac gatctcgtgt ctgagtacca gcagtaccag
6841 gatgccacga ttgaggagga gggcgagttc gacgaggagg agcaatacta gacgcggacg
6901 gggcatttcc cgttcgtcat tagcagtagg taatgaagat gtttgtttct cgtccccttt
6961 ctccttcgtc cttctgtcat tttgttcttt tgtgtttatg ttttgttgtt gttttcttta
7021 attttttttt tcttccacgt ttgtgtacat ccgcgcgcca ctctattcag agagccacgg
7081 atagtagagg aggttggcaa gggtatatga gggacacgcg taccatgatg tgggatgtat
7141 tggggtccct gtctgtcctt acgtgactat gtatgaaccg tcacgtgtaa gatgagctag
7201 tgagatcaac agtacaactc atcaacacgc cttcttctcg ttaaatgtac acaatcttga
7261 tcctccacct ttatgggtcc cattgtttgc ctcttccgct gtgtgagtgc gcctacacgc
7321 acttctcact tcgtaagtgg tggtggcgta agtattgcct aatgttgact ctatgttctc
7381 ctctcctcac cccctcgcgg tgctgatttc tgacagatcc actagttcta gagcggccgc
7441 caccggtgga gctccaattc gccctatagt gagtcgtatt acgcgcgctc actggccgtc
7501 gttttacaac gtcgtgactg ggaaaaccct ggcgttaccc aacttaatcg ccttgcagca

```
7561 catccccctt tcgccagctg gcgtaatagc gaagaggccc gcaccgatcg cccttcccaa
7621 cagttgcgca gcctgaatgg cgaatgggac gcgccctgta gcggcgcatt aagcgcggcg
7681 ggtgtggtgg ttacgcgcag cgtgaccgct acacttgcca gcgccctagc gcccgctcct
7741 ttcgctttct tcccttcctt tctcgccacg ttcgccggct ttccccgtca agctctaaat
7801 cgggggctcc ctttagggtt ccgatttagt gctttacggc acctcgaccc caaaaaactt
7861 gattagggtg atggttcacg tagtgggcca tcgccctgat agacggtttt tcgccctttg
7921 acgttggagt ccacgttctt taatagtgga ctcttgttcc aaactggaac aacactcaac
7981 cctatctcgg tctattcttt tgatttataa gggattttgc cgatttcggc ctattggtta
8041 aaaaatgagc tgatttaaca aaatttaac gcgaatttta acaaaatatt aacgcttaca
8101 atttag
//
```
